# Spontaneous regression of multiple pulmonary metastases accompanied by normalization of serum immune markers following cytoreductive nephrectomy in a patient with clear‐cell renal cell carcinoma

**DOI:** 10.1002/iju5.12252

**Published:** 2020-12-26

**Authors:** Akihito Okazaki, Toshiki Kijima, Philipp Schiller, Natsumi Ishikawa, Hirotaka Fuchizawa, Kohei Takei, Issei Suzuki, Kazumasa Sakamoto, Toyonori Tsuzuki, Takao Kamai

**Affiliations:** ^1^ Department of Urology Dokkyo Medical University Shimotsuga Tochigi Japan; ^2^ Department of Surgical Pathology Aichi Medical University Hospital Nagoya Aichi Japan

**Keywords:** immunity, inflammation, nephrectomy, renal cell carcinoma, spontaneous neoplasm regression

## Abstract

**Introduction:**

The spontaneous regression of metastases, which mostly occurs after surgical resection of the primary tumor, has been described in various malignancies, including renal cell carcinoma. The involvement of the host immune system is currently postulated as the underlying mechanism.

**Case presentation:**

We present a case of metastatic clear‐cell renal cell carcinoma that achieved complete spontaneous regression of multiple pulmonary metastases preceded by normalization of serum immune markers after cytoreductive nephrectomy. The patient remained disease free for 3 years without any systemic therapy, suggesting that postoperative normalization of serum immune markers may indicate recovery of the host immune system, which prevents tumor recurrence.

**Conclusion:**

Monitoring of serum immune markers may be useful to identify patients with recovered immune function and, therefore, may not require systemic therapy. Similarly, the case suggests a potential role of cytoreductive nephrectomy in the contemporary management of metastatic renal cell carcinoma.

**Abbreviations & Acronyms**
CN: cytoreductive nephrectomy
CRP: C‐reactive protein
CT: computed tomography
Hb: hemoglobin
HE: hematoxylin‐eosin
IHC: immunohistochemical
NLR: neutrophil–lymphocyte ratio
PD‐L1: programmed death‐ligand 1
RCC: renal cell carcinoma
sIL‐2R: soluble interleukin‐2 receptor
TIL: tumor‐infiltrating lymphocyte

**Keynote message**

CN could induce spontaneous regression of metastases, which is associated with better survival. The spontaneous regression after CN was preceded by a remarkable decrease in the serum immune markers, such as CRP and sIL‐2R, suggesting the recovery of the host immune system. Thus, monitoring of serum immune markers could help physicians decide optimal treatment modalities for RCC patients. Biomarkers to identify patients who are likely to benefit from CN are strongly warranted in this era of immune checkpoint inhibitors.

## Introduction

The spontaneous regression of metastases has been described in a wide range of malignancies, and RCC is a frequently reported malignancy wherein regressions are observed.^1^, ^2^ The majority of spontaneous regression was observed in cases treated with CN.^3^ The activation of the host immune system has been hypothesized to play a vital role in spontaneous regression. Such an activation of the immune system could result from CN‐mediated depletion of cancer antigens.^2^

Previous studies have demonstrated the prognostic and predictive importance of serum immune markers, such as CRP^4^, ^5^, ^6^, ^7^, ^8^ and sIL‐2R^9^ in metastatic RCC. Here, we present a case of metastatic clear‐cell RCC wherein spontaneous and complete regression of multiple pulmonary metastases, along with normalization of the CRP and sIL‐2R, was observed after CN. The patient remained disease free for 3 years without requiring any systemic therapy, suggesting that the postoperative normalization of serum immune markers may indicate enhanced host immune system, which could prevent tumor recurrence. These findings might help physicians decide an optimal treatment modality for patients with metastatic RCC.

## Case presentation

**Fig. 1:**
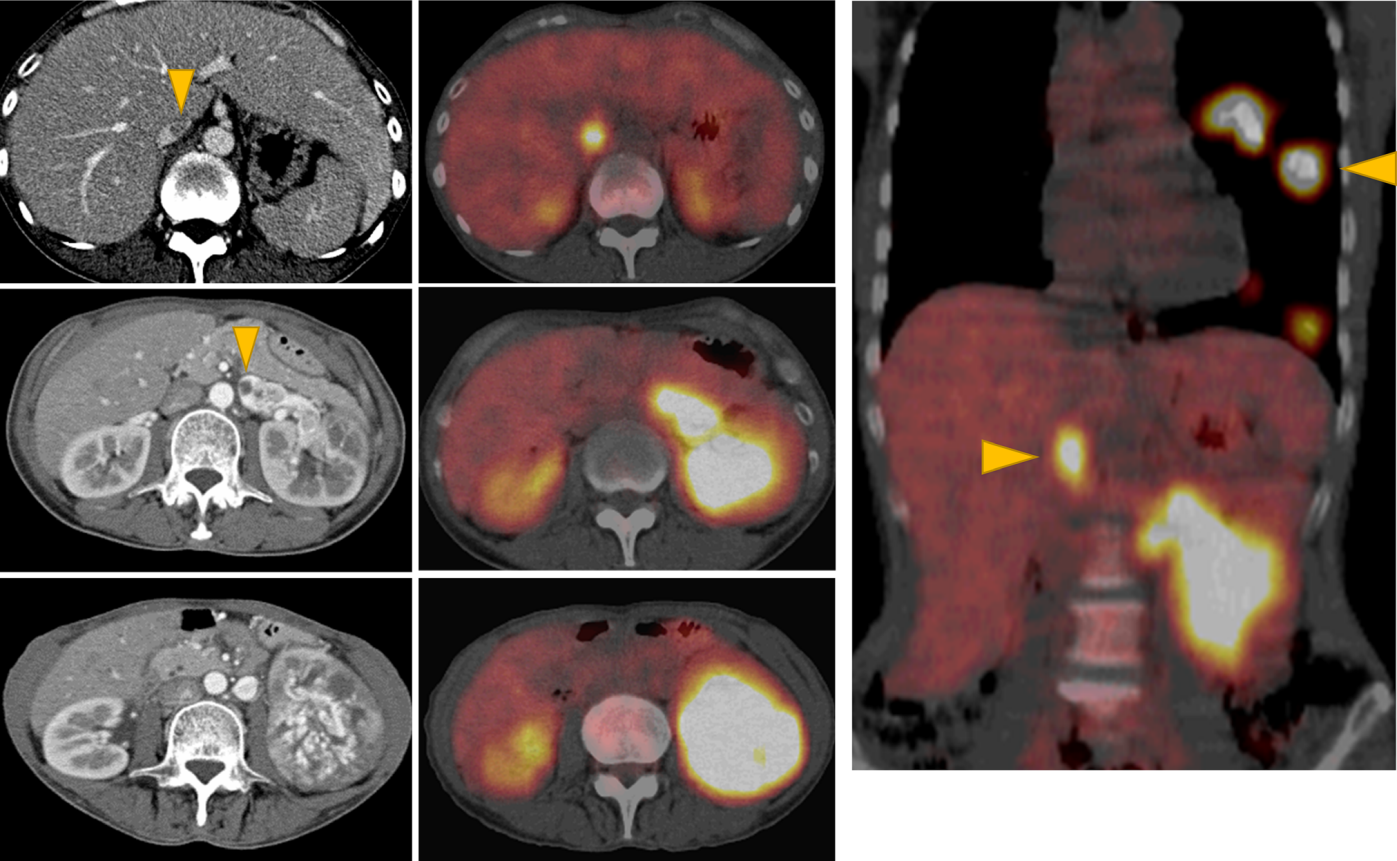
Fluorodeoxyglucose‐positron emission tomography findings revealing the primary tumor in the left kidney, venous thrombus extending into the vena cava, and multiple pulmonary metastases.

A 57‐year‐old woman with no remarkable medical history was admitted to a hospital with symptoms of pyrexia, dry cough, and a weight loss of 5 kg over the last 2 months. CT revealed a left renal tumor, and the patient was referred to our hospital for further investigation and treatment. Contrast‐enhanced CT confirmed the left renal tumor (70 mm in diameter) with a tumor thrombus extending into the inferior vena cava. Similarly, contrast‐enhanced CT revealed the presence of multiple pulmonary metastases. Fluorodeoxyglucose‐positron emission tomography showed abnormal uptake: standardized uptake value 16.0 in the primary tumor, 8.3 in the tumor thrombus, and 18.1 in the pulmonary lesions (Fig. 1) but detected no other distant metastases. Laboratory tests revealed anemia (8.4 g/dL), high neutrophil (8320/μL) and platelet count (54.7 × 10^4^/μL), NLR (9.86), elevation in the sIL‐2R (1360 U/mL, normal range 135–483 U/mL), and a marked elevation in the serum CRP level (33.75 mg/dL; normal range <0.14 mg/dL). Although the patient’s Karnofsky performance status was 80%, she was classified as poor risk based on the International Metastatic RCC Database Consortium risk score.^10^

**Fig. 2:**
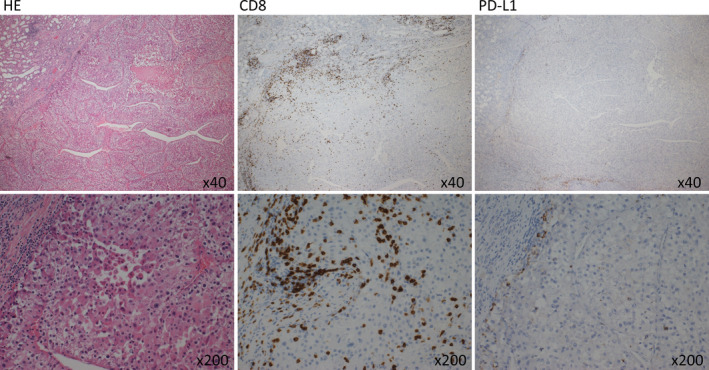
IHC analyses of the primary tumor for CD8 and PD‐L1.

Two weeks after diagnosis, the patient underwent CN with thrombectomy at the left renal vein and vena cava. Pathological diagnosis showed clear‐cell RCC, Grade 3, pT3b. The immune microenvironment of this primary tumor was evaluated by IHC analyses for CD8+ T cell and PD‐L1 (Fig. 2). In HE staining, infiltration of TILs was observed at the tumor margin. These infiltrating cells were confirmed, at least partly, to be CD8+ T cells by IHC. In contrast, the expression of immunosuppressive checkpoint PD‐L1 by tumor cells was extremely weak, suggesting that the primary tumor was recognized and targeted by the host immune system.

**Fig. 3:**
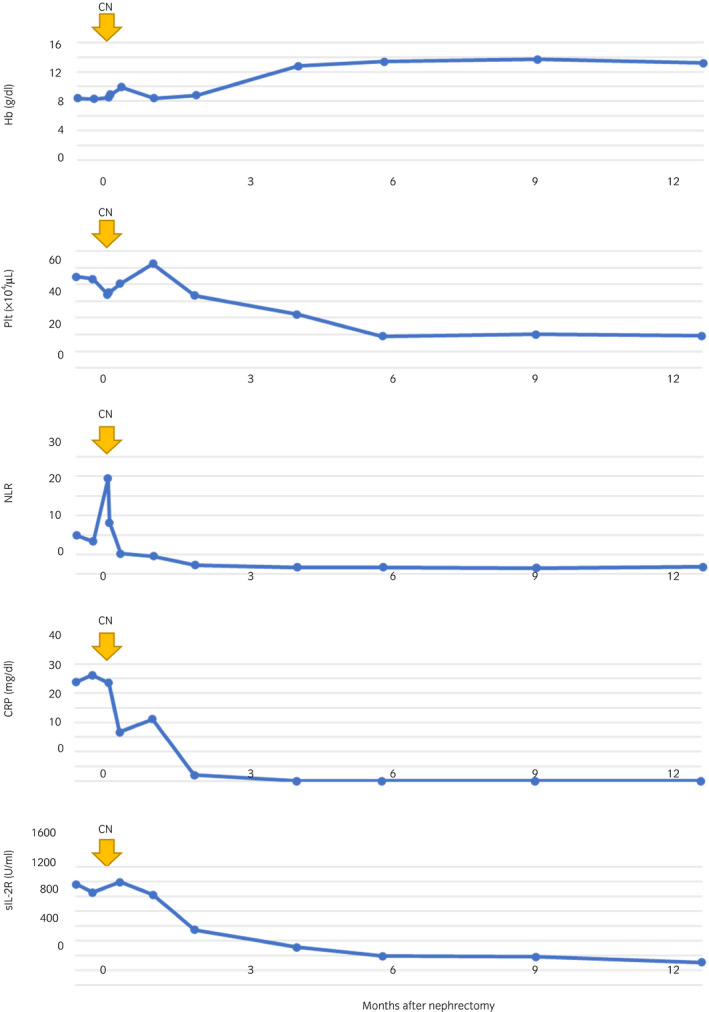
Trends in the serum immune markers following CN.

The patient’s overall well‐being improved after CN, and the serum CRP and sIL‐2R levels after 1 week of surgery decreased to 50% of the presurgical level, which led to us closely monitoring the patient instead of employing immediate systemic therapy. The serum CRP and sIL‐2R levels continued to decrease and reached a normal level at 3 months after CN. In addition, anemia and thrombocytosis gradually improved within 6 months, and NLR immediately decreased after CN (Fig. 3).

**Fig. 4:**
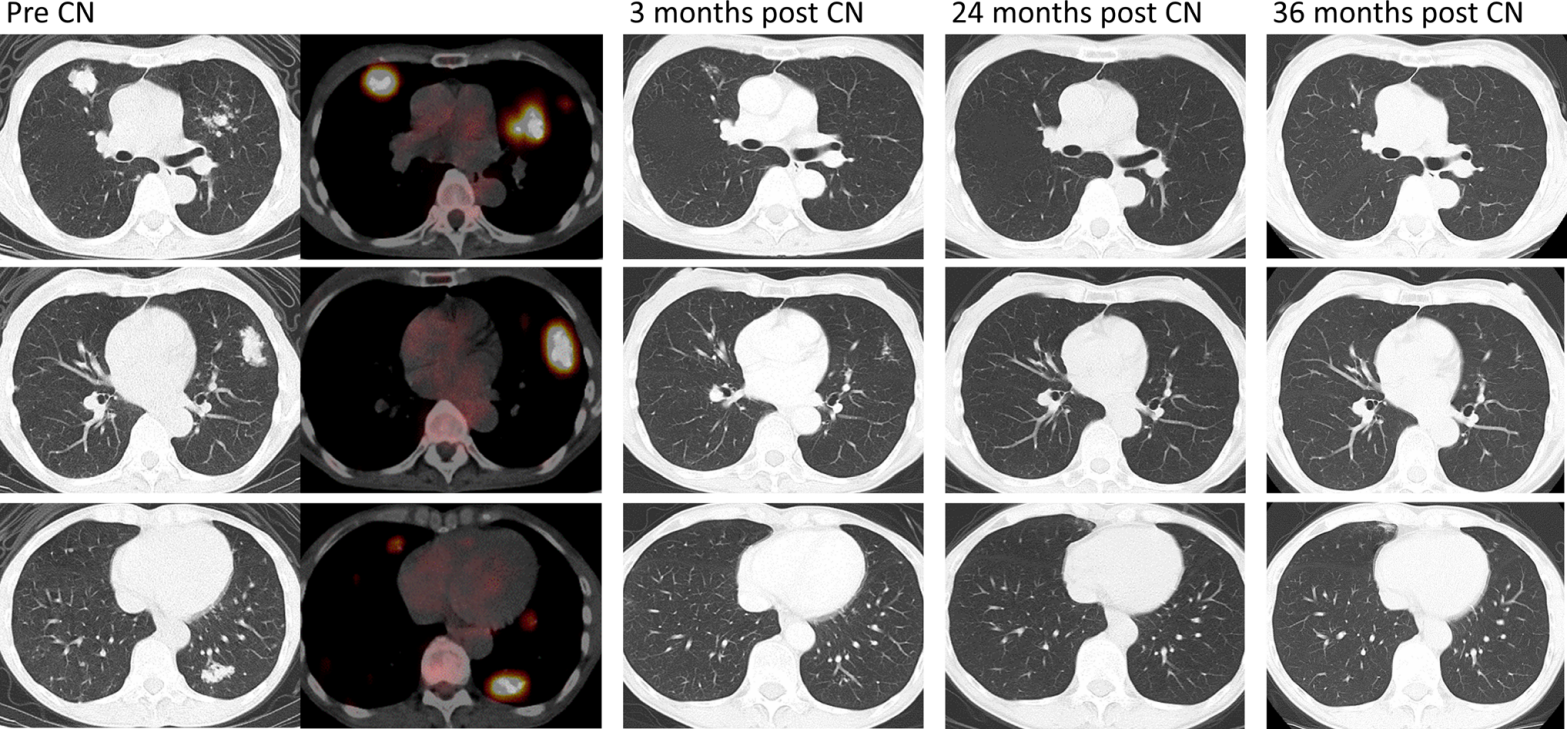
Chest CT scan findings following CN, showing the spontaneous complete regression of multiple pulmonary metastases.

Follow‐up CT scans revealed spontaneously shrinking pulmonary lesions that ultimately disappeared. No development of new lesions was observed, and the patient has been recurrence free for 3 years without requiring any systemic therapies (Fig. 4).

## Discussion

The precise mechanism underlying the spontaneous regression in RCC remains elusive. Most of the reported cases of spontaneous RCC regression are associated with surgical removal of the primary tumor; however, spontaneous regression could occur even after radiation or embolization of the primary tumor.^11^, ^12^ The putative involvement of the immune system is supported by the fact that many patients with spontaneous regression equally developed feverish infection.^13^ In the case of concomitant RCC and autoimmune disease, the spontaneous regression of RCC was accompanied by exacerbation of the latter, further indicating the role of the immune system.^14^ The widely accepted mechanism of spontaneous regression is an enhanced antitumor immune response evoked either by surgery, radiotherapy, tumor necrosis after embolization, autoimmune disease, or infectious diseases.^15^

The prognostic importance of inflammation markers in metastatic RCC patients has been previously reported. In this case, the serum CRP and sIL‐2R levels, which were significantly high during the initial diagnosis, decreased rapidly within a few months after CN, which led to us closely monitoring the patient without systemic therapies. The normalization of the CRP levels after CN was reported to be associated with improved survival in patients with metastatic RCC.^5^, ^8^ Recently, Nakayama *et al*. reported that a high serum CRP level was linked to the immunosuppressive tumor microenvironment in patients with metastatic RCC.^16^ These findings collectively imply that the normalization of the CRP level could indicate recovery of the host immune system.

Interleukin‐2 is a cytokine that regulates the activities of T cells and is released in a soluble form (sIL‐2R) by activated T cells. As interleukin‐2 signaling facilitates not only effector T cells but also inhibitory T cells,^17^ higher sIL‐2R levels could equally represent an immunosuppressive tumor microenvironment. Recently, we reported that preoperative elevation in the sIL‐2R level was associated with poor survival after CN and that patients with reduced sIL‐2R level after CN showed significantly longer survival.^9^ Significant reduction in the levels of sIL‐2R and CRP after CN in this case may indicate recovery from the immunosuppressive tumor microenvironment.

IHC analyses of the primary tumor, in this case revealed the presence of CD8+ TILs in the tumor and extremely low expression of PD‐L1 by the tumor, suggesting that the tumor was the “hot” tumor recognized and targeted by the host immune system.^18^ Although there may be a discrepancy in the immune microenvironment between the primary tumor and metastatic tumors, we speculated that the host immune system might evoke spontaneous regression of metastases in this case.

Although recent prospective studies have questioned the role of CN in the contemporary management of RCC patients,^19^, ^20^ the successful application of CN, in this case, strongly suggests that it can be beneficial in a subset of patients and warrants identification of biomarkers that could specify patients who CN will mostly benefit. Prognostic factors for patients unlikely to benefit from CN include patient characteristics (poor performance status and medical comorbidities), metastatic volume and location (the brain, liver, and bone metastases associated with poor prognosis), and the serum laboratory markers, such as CRP and NLR.^21^ In contrast, presence of tumor thrombus may indicate CN with thrombectomy as surgery may provide a symptomatic benefit by preventing renal, hepatic, or cardiac dysfunction from venous outflow obstruction as tumor thrombus progresses.^22^ In this case, we performed CN considering the good performance status, the presence of tumor thrombus in the vena cava, and the distribution of metastases limited to the lungs. Regarding the serum immune markers, such as CRP and NLR, we have to notice a possibility of changing in these values, following CN, similar to this case. Therefore, clinical decision‐making regarding the performance of CN using these serum immune markers may be inadequate. Instead, postoperative changes in these markers could be useful to decide subsequent therapy after CN.

## Conflict of interest

The authors declare no conflict of interest.
